# Repairing the mitral valve without touching the mitral valve—a novel technique

**DOI:** 10.1093/jscr/rjae845

**Published:** 2025-01-09

**Authors:** Daniel Sitaranjan, Ujjawal Kumar, Fadi Al-Zubaidi, Harry Smith, Sambhavi S Kumar, Stephen Large

**Affiliations:** Department of Cardiac Surgery, Royal Papworth Hospital, Papworth Road, Cambridge Biomedical Campus, Cambridge, Cambridgeshire CB2 0AY, United Kingdom; Department of Cardiac Surgery, Royal Papworth Hospital, Papworth Road, Cambridge Biomedical Campus, Cambridge, Cambridgeshire CB2 0AY, United Kingdom; School of Clinical Medicine, University of Cambridge, Hills Road, Cambridge, Cambridgeshire CB2 0SP, United Kingdom; Department of Cardiac Surgery, Royal Papworth Hospital, Papworth Road, Cambridge Biomedical Campus, Cambridge, Cambridgeshire CB2 0AY, United Kingdom; Department of Cardiac Surgery, Royal Papworth Hospital, Papworth Road, Cambridge Biomedical Campus, Cambridge, Cambridgeshire CB2 0AY, United Kingdom; School of Clinical Medicine, University of Cambridge, Hills Road, Cambridge, Cambridgeshire CB2 0SP, United Kingdom; Department of Cardiac Surgery, Royal Papworth Hospital, Papworth Road, Cambridge Biomedical Campus, Cambridge, Cambridgeshire CB2 0AY, United Kingdom

**Keywords:** left ventricular aneurysm, mitral regurgitation, geometric restoration, ischemic cardiomyopathy

## Abstract

A 44-year-old gentleman presented with severe ischemic cardiomyopathy and mitral regurgitation post-inferior myocardial infarction. Echocardiography and magnetic resonance imaging revealed a dilated left ventricle with a large left ventricular aneurysm (9.3 × 9.5 cm) and associated thrombus. Severe mitral regurgitation due to leaflet tethering and a left ventricular ejection fraction (LVEF) of 25% were also seen. The patient underwent successful aneurysmectomy with patch repair and papillary muscle approximation. Following initial weaning from cardiopulmonary bypass, 6 days of postoperative temporary veno-arterial extracorporeal membrane oxygenation support were required. The patient was subsequently discharged on postoperative day sixteen with improved cardiac function (LVEF of 45%) and trace residual mitral regurgitation, highlighting the efficacy of geometric restoration in addressing such mitral regurgitation, avoiding conventional intervention on the mitral valve itself.

## Introduction

Left ventricular (LV) aneurysms represent a significant mechanical complication of acute myocardial infarction, occurring in up to 35% of transmural infarctions, with 90% having ischaemic aetiology [1, 2]. Surgery remains the mainstay of treatment [3]. Full-thickness infarcts develop granulation tissue at 2–4 weeks, replaced by fibrous tissue at 6–8 weeks, forming an aneurysm [4]. The resultant ventricular geometric distortion leads to secondary mitral regurgitation (MR) through papillary muscle displacement and leaflet tethering [5]. We present a case of successful surgical management by restoring LV geometry, avoiding valve replacement.

## Case report

A 44-year-old gentleman was referred to our cardiothoracic surgery service for consideration of high-risk surgery or transplantation for severe ischemic cardiomyopathy with severe mitral regurgitation following admission to his local hospital for decompensated heart failure. Three months prior to this referral, he had suffered an inferior myocardial infarction. Coronary angiography showed mid-vessel occlusion of the dominant right coronary artery, though the left-sided circulation was unobstructed.

**Figure 1:**
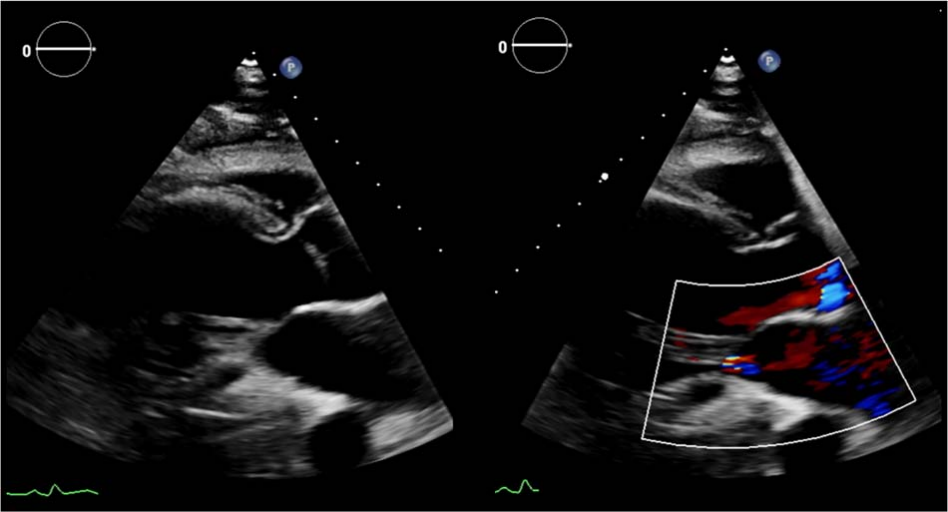
Preoperative echocardiography: large LV aneurysm below the posterior mitral valve leaflet tethering it open in systole resulting in severe functional mitral regurgitation.

Echocardiography (Fig. 1) and cardiac magnetic resonance imaging revealed a dilated LV [left ventricular ejection fraction (LVEF) of 25%] and a large aneurysm (9.3 × 9.5 cm) involving the basal mid-inferoseptum and basal inferolateral wall with evidence of thrombus. These regions were non-viable, with transmural infarction. The mitral valve (MV) leaflets were thin but the tips, especially of the posterior mitral valve leaflet, were tethered and tented towards the LV apex resulting in severe functional MR. The left atrium (LA) was significantly dilated. The patient was admitted for medical management including aggressive diuresis. Following multidisciplinary team (MDT) discussion, surgical intervention was planned (aneurysmectomy with either MV replacement or repair). Mechanical circulatory support and cardiac transplantation were considered and deemed appropriate for rescue therapy.

**Figure 2:**
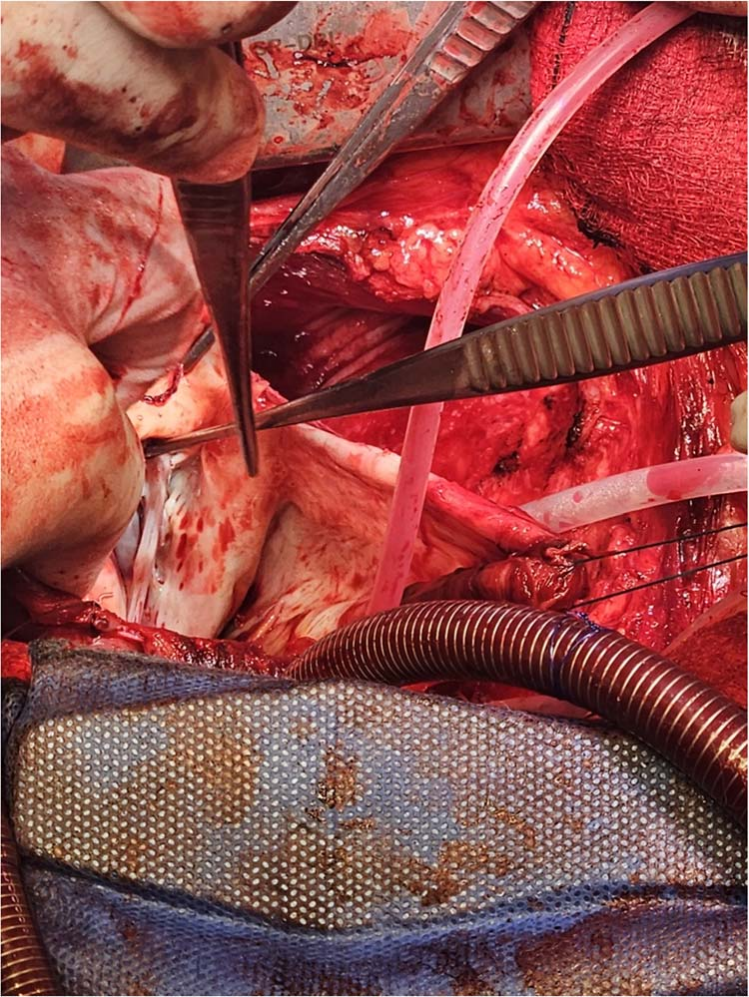
The LV aneurysm was resected down to its neck into healthy ventricular scar tissue. The posterior chordae can be seen from the ventricular defect.

**Figure 3:**
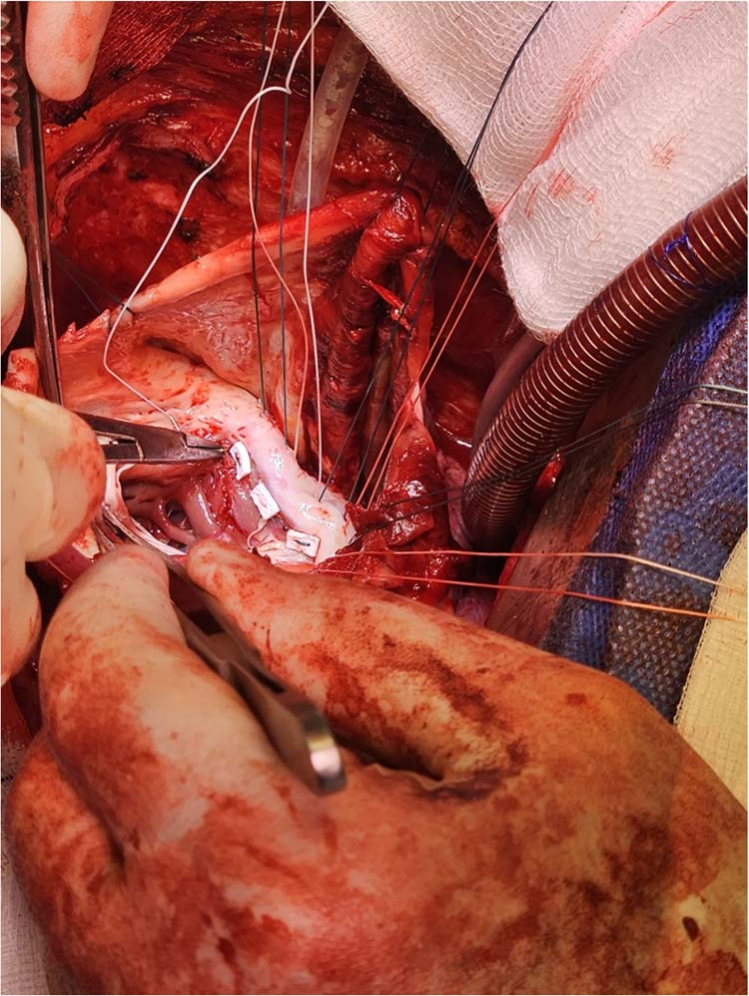
Pledgeted Ethibond sutures were placed circumferentially around the aneurysm mouth.

**Figure 4:**
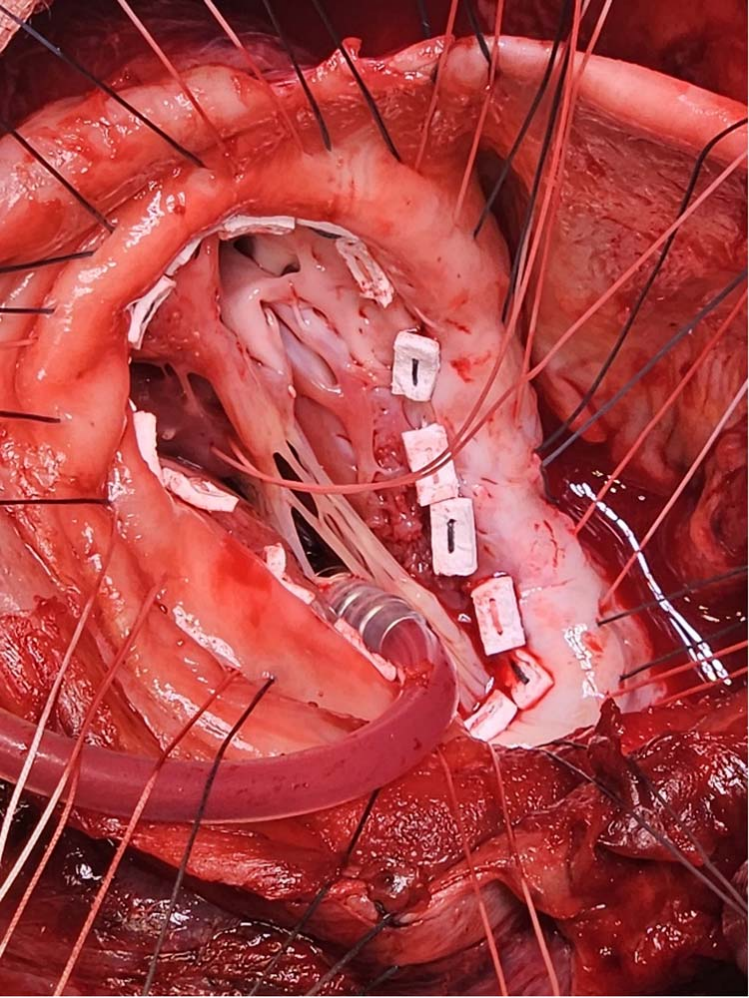
Once the sutures for the patch were placed, the two papillary muscles were identified and another pledgeted suture through the musculotendinous junction was used to approximate them.

**Figure 5:**
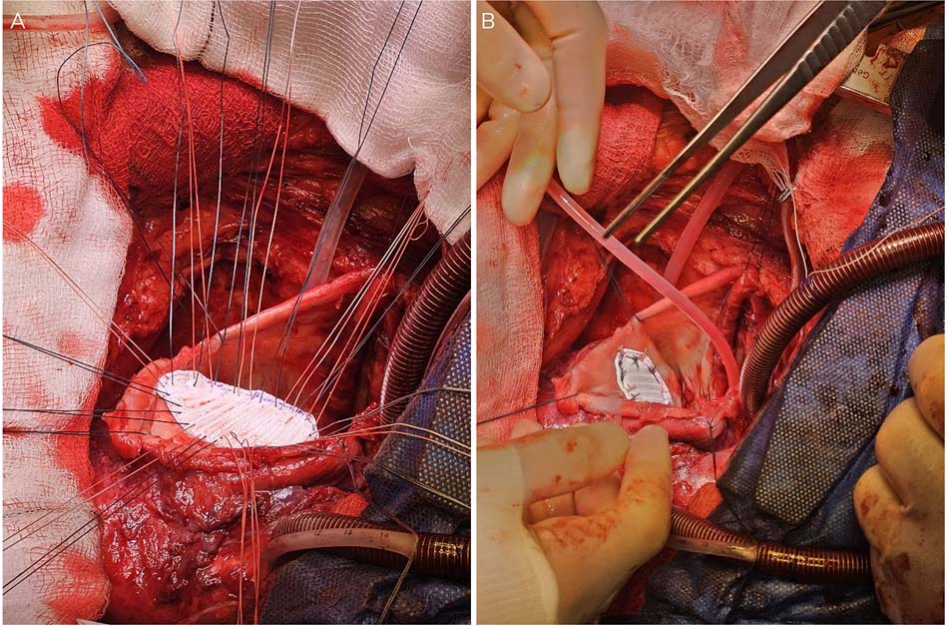
A: The patch was parachuted down and secured. B: Following this the aneurysm sac was closed over the patch for added haemostasis.

Following median sternotomy, cardiopulmonary bypass was instituted (bicaval drainage, ascending aortic return). Antegrade cardioplegia was delivered with aortic root venting and bicaval snaring. Dense adhesions surrounded the aneurysm, which had a thin wall, measured ⁓15 cm from LV apex to mitral annulus, and contained around 150 g of clot. The posterior mitral leaflet appeared retracted, preventing coaptation due to LV stretching. The aneurysm was resected (Fig. 2), and the LV wall repaired with a 5 × 3 cm patch, restoring normal LV size (Fig. 3). The musculotendinous junctions of the anterior and posterior papillary muscles were approximated using pledgeted 3–0 polypropylene sutures (Fig. 4). The patch repair was completed (Fig. 5), and the overlying sac oversewn for haemostasis.

**Figure 6:**
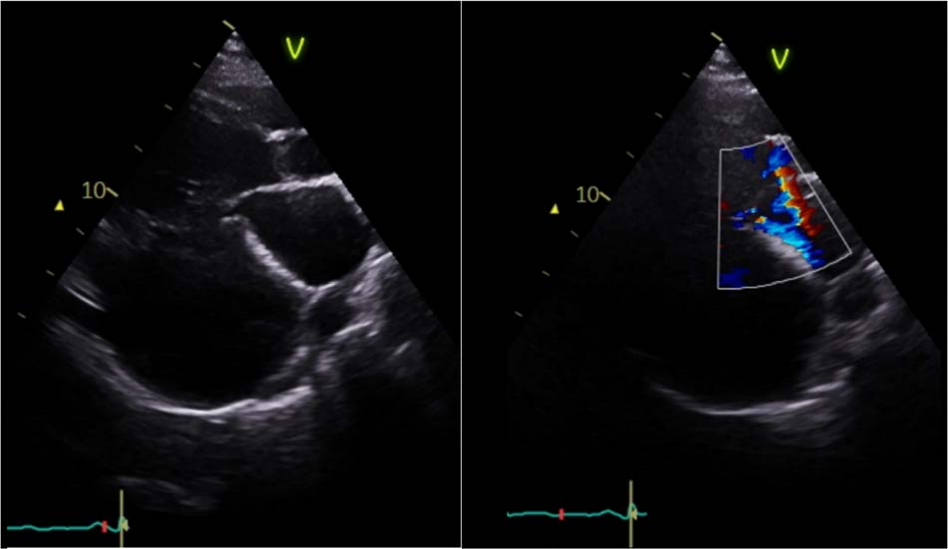
Postoperative echocardiography: restoration of mitral valve leaflet coaptation and LV geometry, resulting in trivial regurgitant jet.

The heart was deaired and pacing wires placed. Despite successful initial weaning from cardiopulmonary bypass (CPB) with support (dopamine, adrenaline, noradrenaline), repeat pharmacological intervention was necessary. Central veno-arterial extracorporeal membrane oxygenation (VA-ECMO) was established by exchanging bicaval cannulae for a two-stage venous drainage cannula and utilizing the existing aortic cannula. Flows of 4–5 L/min were maintained, and the heart continued to eject with aortic valve opening. The patient was transferred to intensive care in a stable condition and recovered on ECMO for 6 days before decannulation and chest closure 48 h later. Following medical optimisation, he was discharged on postoperative day sixteen. Postoperative echocardiography (Fig. 6) revealed LV improvement (LVEF 45%) with a competent MV (trace regurgitation, no stenosis). At 1-year follow-up, the patient was recovering well without complications with preserved ejection fraction and mild residual MR.

## Discussion

LV aneurysms can disrupt cardiac geometry and mechanics, increasing interpapillary muscle distance and impairing MV function [1, 5]. Clinical sequelae can be severe [6], as seen in our patient who presented with decompensated heart failure, severe MR, and significant thrombus. Secondary MR results from unbalanced leaflet forces due to LV and LA geometric distortion. Mechanisms of ischemic MR include dilated cardiomyopathy, where a dilated LV can cause poor leaflet coaptation and inferobasal infarction leading to posterior leaflet tethering and aneurysm formation [5]. Additionally, atrial functional MR in patients with atrial fibrillation can lead to LA enlargement and mitral annular dilatation. Papillary muscle rupture can also occur, leading to leaflet prolapse.

Surgical decision-making in such cases is complex and current evidence supports individualized decision-making. Repair or replacement are established treatments for ischaemic MR. Clinical studies have shown that whilst repair is associated with lower perioperative mortality, replacement may provide better long-term outcomes with lower risks of recurrence [7]. However, a randomized trial of 251 patients found no difference in LV remodelling between repair and replacement groups at 12 months, challenging previous paradigms [8]. International guidelines reflect this complexity. American guidelines suggest considering valve replacement in the presence of basal aneurysm/dyskinesis, significant leaflet tethering, or moderate/severe LV remodelling (left ventricular end-diastolic diameter (LVEDD) > 65) [9]. Otherwise, MV repair with an undersized complete rigid ring should be considered. Similarly, European guidelines recommend repair in patients without advanced LV remodelling, or replacement when there is a risk of repair failure [10].

Recent interest has focused on chord-sparing replacement [11] and subvalvular interventions [12], with evidence that papillary muscle approximation compared to reduction annuloplasty alone shows long-term benefits for LV remodelling [13]. In this case, the significant ventricular remodelling and basal aneurysm suggested that geometric restoration might sufficiently restore MV function, thus avoiding complications of valve intervention, such as prosthetic valve-related events or repair failure.

This case demonstrates successful treatment of severe ischaemic MR through geometric restoration of the LV without direct MV intervention. Surgery had two key elements to address the underlying pathophysiology of the MR: LV aneurysmectomy with patch repair and papillary muscle approximation. These interventions successfully restored LV geometry whilst restricting further dilatation. The improvement in LVEF (25%–45%) and restoration of valve competency support the efficacy of this approach in carefully selected cases. The need for temporary VA-ECMO support highlights the complexity of such cases and the value of mechanical circulatory support as a bridge to recovery.

Our experience suggests that in patients with ischaemic MR secondary to LV aneurysm, restoration of ventricular geometry combined with papillary muscle approximation may be sufficient to address the MR without direct valve intervention. This may be particularly valuable in younger patients, where avoiding valve intervention could have significant long-term benefits. Future research should focus on identifying specific anatomical and functional parameters to assess which patients would benefit most from this approach versus conventional valve surgery. Long-term follow-up studies would be valuable to assess the durability of this technique and its impact on patient outcomes.
